# Case of Rupture of the Ductus Communis Choledochus

**Published:** 1849-07

**Authors:** W. B Parker

**Affiliations:** Moravia, N. Y.


ART. III.—*Case of rupture of the Ductus Communis Choledochus.* By W. B. PARKER, M. D., Communicated in a letter to Prof. C. A. LEE.

DEAR SIR:—

According to my promise, I give you briefly the history of the case of rupture of the common duct, and the subsequent examination. During my stay in Kelloggsville, I called at a harness maker's shop, February 9th.— I there saw the patient for the first time: he was at work at his trade, (harness making,) he complained of a cramp like pain in the gastic region, and wanted to know of me what it indicated. By his description I concluded it to be of a nervous character, and told him so. The pain would leave him, when he left his work, and recur on his resuming it again. I did not make any prescription for him, as he thought he did not need it.— I heard nothing more from him until the Tuesday following, when I heard that he had had the night before, a severe attack of the same cramp-like pain, but more intense and lancinating. It (the pain) would leave him in a short time, then recur with greater violence. He called on a Botanic or Eclectic practitioner, who called the disease at first spasmodic jaundice; a short time afterwards he called it enteritis: but another change came over him, and then he called it bilious colic. His treatment was first an emetic, which increased the patient's misery. This was on Monday. Tuesday morning cathartics were administered with no good effect. Pain increased and spread over the abdomen. In the after part of the day croton oil was given in connection with senna injections. An evacuation was at last produced, but the patient grew worse. In the evening I called to see him, and found him laboring under an attack of acute peritonitis. The symptoms were all well marked with one exception, and that was an absence of pain, or rather no increase of pain on pressure over the abdomen, but there was a cause for that, as dissection subsequently showed:—there was about an inch of adipose tissue between the integuments and muscles of the abdomen. There was one place that pressure would increase the pain, and that was just above the centre of the Umbilical region ; and in that place a hernia was found, but it never could, under ordinary circumstances, become strangulated, as the neck of the sac was as large as the sac itself. Tuesday night, at the hour of twelve, the patient died, after an illness of twenty-eight hours. The examination was made eight hours after death. We first found an old umbilical hernia as I have before stated. The peritoneal cavity was filled with serum.— The intestines were everywhere agglutinated together, and covered with pus. There were also on their surfaces gangrenous patches. The cœcum was inflamed over its whole surface, and covered with pus and bile. The ascending portion of the colon presented the same appearance. There was also a very offensive odor (that of putrid bile) on cutting into the cavity of the abdomen. The Omentum was hard and brittle, breaking like brittle wood. It was also very much thickened. Its surface was covered with a calcareous matter, which turned the edge of the scalpel as if it were drawn across a stone. The right lobe of the liver was enlarged, indurated, and very dark colored, nearly black. It was adherent to the diaphragm, ribs, and the duodenum; the *adhesions to the latter had been torn up, and had involved the common duct which was torn across about half way between the union of the Hepatic and cystic ducts, and its entrance into the duodenum.* Left lobe of liver natural in size, and healthy. Spleen enlarged, stomach healthy. Right kidney natural, left enlarged. Pancreas not examined. Mesenteric glands were enlarged, and felt as though they contained tuberculous matter. The coats of the urinary bladder thickened and indurated. The gall bladder was empty, its contents had escaped into the cavity of the peritoneum, and had produced excessive inflamation. The adhesions of the right lobe of liver were of long standing, and probably would not have been torn up had not the patient gone to work at his trade, after doing nothing for some time previous to his coming to Kelloggsville. He had been there but three or four days previous to his illness. I think his illness was caused by his raising his arms and thereby drawing on the muscles of the ribs, (the intercostals,) by the previous action of the pectoral muscles, which had a tendency to draw the liver up with the ribs, and thereby detaching the adhesions on the under side of that viscus. This was probably the first cause of the cramp-like pains of which he complained, and the emetic which he took probably occasioned the latter trouble, that is, the laceration of the ductus communis choledochus, and thereby the patient's death.

MORAVIA, N. Y. May 29th, 1849.

